# Allelic variants of breast cancer susceptibility genes *PALB2* and *RECQL* in the Latvian population

**DOI:** 10.1186/s13053-019-0116-6

**Published:** 2019-07-03

**Authors:** Philip Hilz, Reicela Heinrihsone, Lukas Alexander Pätzold, Qi Qi, Genadijs Trofimovics, Linda Gailite, Arvids Irmejs, Janis Gardovskis, Edvins Miklasevics, Zanda Daneberga

**Affiliations:** 1 Institute of Oncology, Riga Stradins University, Dzirciema street 16, Riga, LV-1007 Latvia; 2 Department of Integrative Oncology, Fudan University Shanghai Cancer Center, Shanghai, 200032 People’s Republic of China; 3 Department of Surgery, Riga Stradins University, Dzirciema street 16, Riga, LV-1007 Latvia; 4 Scientific Laboratory of Molecular Genetics, Riga Stradins University, Dzirciema street 16, Riga, LV-1007 Latvia; 5 Present address:Center for Anaesthesiology and Intensive Care Medicine, Martinistreet 52, Building Ost 10, 2.OG, 20246 Hamburg, Germany

**Keywords:** Breast cancer, *PALB2*, *RECQL*, Moderate risk allele, Low frequency allele

## Abstract

**Background:**

Large-scale case control studies revealed a number of moderate risk - low frequency breast cancer alleles of the *PALB2* and *RECQL* genes. Some of these were reported as founder variants of Central and Eastern Europe. Based on highly similar founder variant spectra of the *BRCA1* in Poland and Latvia, we decided to test the frequency of other common variants of moderate breast cancer risk — c.509_510delGA (rs515726124) and c.172_175delTTGT (rs180177143) of the *PALB2* gene and c.1667_1667+3delAGTA variant of the *RECQL* gene in a breast cancer case-control series from Latvia to better understand the role of genes in susceptibility to breast cancer and their clinical significance.

**Methods:**

The case-control study was performed based on an unselected breast cancer case group of 2480 women and a control group, including 1240 voluntary, to our knowledge unrelated, female donors without reported oncological disease.

**Results:**

The calculated frequency for c.509_510delGA of the *PALB2* gene in the case group is 0.35 and 0.00% in the control group, with respective relative risk (RR) 7.18 (CI 95% 0.37–138.75; *p* = 0.19). As for the *PALB2* c.172_175delTTGT variant, the frequency in the case group of our study is 0.04%. In the control group of our study all individuals were homozygous for the wild-type allele, which lead to calculated RR = 1.50 (CI 95% 0.06–36.83; *p*-value = 0.80). There were no carriers of the *RECQL* variant c.1667_1667+3delAGTA identified in our case group and 2 heterozygotes were identified in the control group. The calculated RR = 0.26 (CI 95% 0.01–5.33; *p*-value = 0.38).

**Conclusion:**

Results obtained for the *PALB2* gene variants are able to supplement evidence on the allele frequency in breast cancer patients from the region of Central and Eastern Europe. Based on our results we cannot confirm the contribution of the *RECQL* variant c.1667_1667+3delAGTA allele to breast cancer development.

## Background

High penetrance breast cancer susceptibility genes — *BRCA1* and *BRCA2* are responsible only for 5–10% of familial breast cancers [1]. Large-scale case control studies revealed a number of so-called moderate risk breast cancer alleles. Those can further be subdivided into high penetrance-low frequency (i.e. *TP53*, *SKT-11*, *PTEN* and *CDH1*) and moderate penetrance-low frequency (i.e. *ATM*, *CHEK2*, *RAD51* and *PALB2*) alleles. The prevalence of moderate penetrance variants usually varies among different populations and requires statistical evidence from association studies of breast cancer cases and population-matched controls [1, 2]. Lately the *RECQL* gene is also considered a breast cancer susceptibility gene [3–5]. Many of the genetic variants of the mentioned genes still lack clinical validation as breast cancer susceptibility polymorphisms.

The *PALB2* (Partner and Localizer of *BRCA2*) gene encoding a protein is involved in the intranuclear stabilization and localization of the BRCA2 protein and supports some of its functions in homologous recombination and double strand break repair. Monoallelic variants and a heterozygous state are proven to be associated with a high risk for breast cancer development, almost comparable to that of *BRCA2* [6]. With a frequency ranging from 0.5 to 1.0% in general breast cancer populations the truncating allelic variants c.509_510delGA (rs515726124) and c.172_175delTTGT (rs180177143) have recently been discussed in the region of Central and Eastern Europe - Poland, Belarus, Germany and Russia [7, 8]. The first report of c.509_510delGA allele (*PALB2*) frequency identified 4 of 648 (0.6%) unrelated familial breast cancer patients from central Poland [9]. Noskowicz and colleagues identified the c.509_510delGA allele in 3/1008 (0.3%) German breast cancer patients, 2/994 (0.2%) Russian breast cancer patients and 5/1922 (0.3%) Belarusian breast cancer patients [7]. In 2015 another group of researchers from Poland published a large cohort analysis which showed frequencies of 0.61% for the c.509_510delGA allelic variant and 0.32% for the c.172_175delTTGT, respectively [8]. Clinical significance of the c.509_510delGA allelic variant based on the ClinVar database is pathogenic; for the c.172_175delTTGT allelic variant - conflicting interpretations of pathogenicity [10].

As for most established breast cancer susceptibility genes, *RECQL* (*RECQ1*) encodes a protein, functioning in DNA repair. As part of the RecQ DNA helicase family, its role is thought to be DNA unwinding, replication fork stabilisation and assisting branch migration and strand annealing [11]. The allelic variant c.1667_1667+3delAGTA in the *RECQL* gene is described as a founder mutation in the studied population from Poland, with a frequency of 0.23% in the group of unselected breast cancer cases and 0.04% in the control group with OR = 5.4 [5]. Based on in silico prediction this allelic variant leads to the splice site change and to changes in protein level p.K555delinsMYKLIHYSFR [12]. Recent publications on results for c.1667_1667+3delAGTA screening in breast cancer patients and controls from Eastern European and Central European populations have tried to clarify the exact magnitude of the risk. The results of a breast cancer case-control series from Belarus and Germany revealed OR = 1.23 (95% CI 0.44–3.47, *p*-value = 0.69) [13]. In the study on the *RECQL* variants in 427 women affected with breast or ovarian cancer from southwest Poland and West Ukraine no heterozygous carrier of the c.1667_1667 + 3delAGTA variant was found [14]. Altogether, the allelic variant might be considered a moderate-risk breast cancer susceptibility allele. Clinical significance of the variant is uncertain.

Based on highly similar founder variant spectra of *BRCA1* in Poland and Latvia, we decided to test the frequency of other common variants of moderate breast cancer risk —c.509_510delGA (rs515726124) and c.172_175delTTGT (rs180177143) of the *PALB2* gene, and the c.1667_1667+3delAGTA variant of the *RECQL* gene. The breast cancer case-control series from Latvia was genotyped to gain information on their frequency as well as a better understanding of the role of genes in susceptibility to breast cancer and their clinical significance.

## Methods

The case-control study was performed based on an unselected breast cancer case group, including women who met the criteria for breast cancer (C-50) according to the International Classification of Diseases (ICD-10), and a control group, including voluntary, to our knowledge unrelated, female donors without reported oncological disease. The case group consisted of 2480 women and the control group consisted of 1240, included into the study between 2008 and 2015. Ages in the breast cancer group ranged from 24 to 86 years. Ages of the gender-matched controls ranged from 18 to 92 years. Informed consent was obtained from all individual participants included in the study.

The allelic variant c.509_510delGA of the *PALB2* gene was analysed using the restriction fragment length polymorphism (RFLP) method. For amplification of a 322 bp long target region of exon 4 forward primer 5′- AGTCCTTTAACCCTGGAGAT-3′ and reverse primer 5′- GGTTCTGGAGAATCTGGAAG-3′ were used. Restriction endonuclease DdeI (New England Biolabs, Ipswich, Massachusetts, USA) recognising the allelic variant sequence was applied. Acquired fragments were separated in 2.5% agarose gel.

The target region of the *RECQL* allelic variant c.1667_1667 + 3delAGTA was amplified with forward primer 5′- TATTTCAGTCTGCTAGCTTAGTT-3′ and reverse primer 5′- TAATAGAAGCAGAGATTTCCATCAT-3′, resulting in a 540 bp fragment which was subjected to restriction endonuclease MspC1 (BIORON GmbH, Ludwigshafen, Germany) to cut DNA in the control site and in the case of the wild type allele, resulting in four fragments. RFLP was analysed in 2.5% agarose gel.

For analysis of the *PALB2* gene allelic variant c.172_175delTTGT the target PCR product was subjected to the denaturing high performance liquid chromatography (DHPLC) method, according to the manufacturers protocol (Transgenomic Inc., Omaha, NE, USA). All primers for amplification of target gene regions were designed using NCBI Primer BLAST [15].

All protocols applied to analyse allelic variants were verified by Sanger sequencing according to the manufacturers protocol (Life Technologies, Carlsbad, CA, USA). Statistical analyses were performed using MedCalc for Windows, version 15.0 (MedCalc Software, Ostend, Belgium).

## Results

Three allelic variants of the *PALB2* and the *RECQL* genes were analysed in the case and control groups.

The calculated frequency for c.509_510delGA of the *PALB2* gene was in line with results from the Belarusian and German populations, but lower than those reported in the Polish population, with respective relative risk (RR) 7.18 (CI 95% 0.37–138.75; *p* = 0.19) (Table 1). As for the *PALB2* c.172_175delTTGT variant, the frequency in the case group of our study is significantly lower, as in the published data from Poland. In the control group of our study no heterozygous carriers were detected (Table 1), which lead to calculated RR = 1.50 (CI 95% 0.06–36.83; *p*-value = 0.80). There were no carriers identified in our case group and 2 heterozygotes were identified in the control group (Table 2). The calculated RR = 0.26 (CI 95% 0.01–5.33; *p*-value = 0.38).

**Table 1:** Frequency of the *PALB2* gene allelic variants in breast cancer patients and controls from Easter European and Central European populations

Population	Polish [9]	Polish [8]	German [7]	Russian [7]	Belarusian [7]	Latvian (present study)
Case group						
c.509_510delGA	4/648 (0.60%)	76/12529 (0.61%)	3/1008 (0.30%)	2/994 (0.20%)	5/1992 (0.30%)	3/869 (0.35%)
c.172_175delTTGT	N/A	40/12529 (0.32%)	N/A	N/A	N/A	1/2479 (0.04%)
Control group						
c.509_510delGA	1/1310 (0.08%)	10/4702 (0.21%)	0/2827 (0.00%)			0/891 (0.00%)
c.172_175delTTGT	N/A		N/A	N/A	N/A	0/1240 (0.00%)

**Table 2:** Frequency of the *RECQL* gene allelic variant in breast cancer patients and controls from Eastern European and Central European populations

Population	Polish [5]	Polish and Ukrainian [14]	German and Belarusian [13]	Latvian (present study)
Case group				
c.1667_1667+3delAGTA	30/13136 (0.23%)	0/427 (0.00%)	9/2596 (0.35%)	0/715 (0.00%)
Control group				
c.1667_1667+3delAGTA	2/4702 (0.04%)	N/A	6/2132 (0.28%)	2/916 (0.22%)

Variant c.1667_1667+3delAGTA of the *RECQL* gene reported as founder variant in the Polish population was also analysed in our study. There were no carriers identified in our case group and 2 heterozygotes were identified in the control group (Table 2). The calculated RR = 0.26 (CI 95% 0.01–5.33; *p*-value 0.38).

## Discussion

The *PALB2* gene variants have been identified as breast cancer susceptibility alleles by previously published research from Eastern and Central Europe, as well as the ClinVar database which has classified the c.509_510delGA allelic variant as pathogenic. Our results are in concert with previously published findings and indicate c.509_510delGA as a low-frequency variant in the tested population, which should not be overlooked as a causative variant in familial breast cancer. The *PALB2* c.172_175delTTGT variant was found at very low frequency in the case group. Due to the small number of analysed cases, the results cannot contribute to the interpretation of variant pathogenicity. However, allele frequency is comparable to that of neighbouring populations, excluding Poland.

The results for *RECQL* variant c.1667_1667+3delAGTA in the control group show comparable frequency to that reported by Bogdanova et al. [13], and much higher as reported by Cybulski et al. [5]. We did not detect any heterozygotes in the case group. Based on the ExAC database, the frequency of c.1667_1667+3delAGTA is 0.035% [16]. The control group results of Cybulski et al. are in line with the ExAC database; however, our study results and those of Bogdanova et al. yielded higher allele frequencies in the control group. Interestingly, in the German and Belarusian populations, the difference between allele frequency in case and control groups is minimal. Taking into account the broad age range of our control group, we cannot exclude the possibility of positive cases developing disease in later life.

The combined data of the *RECQL* variant c.1667_1667+3delAGTA frequency in case-control studies from Eastern and Central European populations cannot support assigning this variant to the group of moderate risk alleles. More case-control study results from the neighbouring populations are needed to confirm the impact of this variant on the development of breast cancer.

## Conclusion

Results obtained for the *PALB2* gene variants are able to supplement evidence on the allele frequency in breast cancer patients from the region of Central and Eastern Europe. Based on our results we cannot confirm the contribution of the *RECQL* variant c.1667_1667+3delAGTA allele to breast cancer development.

## Data Availability

The datasets used and/or analysed during the current study are available from the corresponding author on reasonable request.

## References

[1] Apostolou P, Fostira F (2013). Hereditary breast cancer: the era of new susceptibility genes. Biomed Res Int.

[2] Hollestelle A, Wasielewski M, Martens JW, Schutte M (2010). Discovering moderate-risk breast cancer susceptibility genes. Curr Opin Genet Dev.

[3] Banerjee T, Brosh RM (2015). RECQL: a new breast cancer susceptibility gene. Cell Cycle.

[4] Akbari MR, Cybulski C (2015). RECQL: a DNA helicase in breast cancer. Oncotarget..

[5] Cybulski C, Carrot-Zhang J, Kluźniak W, Rivera B, Kashyap A, Wokołorczyk D (2015). Germline RECQL mutations are associated with breast cancer susceptibility. Nat Genet.

[6] Couch FJ, Hart SN, Sharma P, Toland AE, Wang X, Miron P (2015). Inherited mutations in 17 breast cancer susceptibility genes among a large triple-negative breast cancer cohort unselected for family history of breast cancer. J Clin Oncol.

[7] Noskowicz M, Bogdanova N, Bermisheva M, Takhirova Z, Antonenkova N, Khusnutdinova E (2014). Prevalence of PALB2 mutation c.509_510delGA in unselected breast cancer patients from central and Eastern Europe. Familial Cancer.

[8] Cybulski C, Kluźniak W, Huzarski T, Wokołorczyk D, Kashyap A, Jakubowska A (2015). Clinical outcomes in women with breast cancer and a PALB2 mutation: a prospective cohort analysis. Lancet Oncol.

[9] Dansonka-Mieszkowska A, Kluska A, Moes J, Dabrowska M, Nowakowska D, Niwinska A (2010). A novel germline PALB2 deletion in polish breast and ovarian cancer patients. BMC Med Genet..

[10] Landrum MJ, Lee JM, Benson M, Brown GR, Chao C, Chitipiralla S (2017). ClinVar: improving access to variant interpretations and supporting evidence. Nucleic Acids Res.

[11] Pike ACW, Gomathinayagam S, Swuec P, Berti M, Zhang Y, Schnecke C (2015). Human RECQ1 helicase-driven DNA unwinding, annealing, and branch migration: insights from DNA complex structures. Proc Natl Acad Sci U S A.

[12] Schwarz JM, Cooper DN, Schuelke M, Seelow D (2014). MutationTaster2: mutation prediction for the deep-sequencing age. Nat Methods.

[13] Bogdanova N, Pfeifer K, Schürmann P, Antonenkova N, Siggelkow W, Christiansen H (2017). Analysis of a RECQL splicing mutation, c.1667_1667+3delAGTA, in breast cancer patients and controls from Central Europe. Familial Cancer.

[14] Nguyen-Dumont T, Myszka A, Karpinski P, Sasiadek MM, Akopyan H, Hammet F (2018). FANCM and RECQL genetic variants and breast cancer susceptibility: relevance to South Poland and West Ukraine. BMC Med Genet.

[15] Ye J, Coulouris G, Zaretskaya I, Cutcutache I, Rozen S, Madden TL (2012). Primer-BLAST: a tool to design target-specific primers for polymerase chain reaction. BMC Bioinf.

[16] Lek M, Karczewski K, Minikel E, Samocha K, Banks E, Fennell T (2016). Analysis of protein-coding genetic variation in 60,706 humans. Nature..

